# Autospreader Flaps in Closed Rhinoplasty: Our Technique and Long-Term Results

**DOI:** 10.7759/cureus.66458

**Published:** 2024-08-08

**Authors:** Raymond Challita, Deoda Maassarani, Nancy Zeaiter, Joseph Sfeir, Charbel B Aoun, Elie Moukawam, Nina Rossa Haddad, Diala El Chbib, George Ghanime, Ziad Sleiman

**Affiliations:** 1 Plastic and Reconstructive Surgery, Grenoble Alpes University Hospital, Grenoble, FRA; 2 Plastic and Reconstructive Surgery, Faculty of Medicine, Lebanese University, Beirut, LBN; 3 Plastic Surgery, Lebanese Hospital Geitaoui University Medical Center (UMC), Aschrafieh, LBN; 4 Plastic and Maxillofacial Surgery Department, Al Zahraa University Medical Hospital, Beirut, LBN; 5 Internal Medicine, Lebanese University, Beirut, LBN; 6 Obstetrics and Gynecology, Faculty of Medicine, Lebanese University, Beirut, LBN; 7 Plastic and Reconstructive Surgery, Lebanese Hospital Geitaoui University Medical Center (UMC), Aschrafieh, LBN; 8 Plastic and Reconstructive Surgery, Lebannese University, Beirut, LBN

**Keywords:** spreader grafts, spreader flaps, internal nasal valve, nose score, endonasal rhinoplasty

## Abstract

Introduction: Rhinoplasty is a common and complex surgical procedure. Respiratory and aesthetic dissatisfaction are major causes of revision surgeries. Multiple techniques were described to reconstruct the middle nasal vault and improve functional outcomes. One of these techniques is the use of autospreader flaps. These flaps were constantly modified by different surgeons. In our practice, we use a modified technique of autospreader flaps in closed rhinoplasty. Neither upper lateral cartilage scoring nor suture fixation to the septum was done.

Methods: We conducted a retrospective study on 183 patients, analyzing revision rates and long-term functional results using the NOSE scale. Data analysis was done using IBM Corp. Released 2019. IBM SPSS Statistics for Windows, Version 26.0. Armonk, NY: IBM Corp.

Results: Long-term results showed satisfactory aesthetic outcomes with low revision rates (13.6%). Concerning the NOSE score, it was completed by 87 of the 183 patients, yielding a response rate of 47.5%. A mean NOSE score of 18.1 +/- 21.1 at 4.4 years of follow-up was obtained.

Conclusion: Autospreader flaps offer simplicity, reproducibility, and effectiveness in closed rhinoplasty. It represents a valuable option for selected patients, especially in populations with high dorsal reduction surgery demand.

## Introduction

Rhinoplasty is a common aesthetic surgical procedure. According to the 2022 International Society of Aesthetic Plastic Surgery (ISAPS) global survey, it ranks among the top 10 cosmetic surgical procedures performed worldwide [1]. It is a complex procedure with variable revision rates, as reported in the literature [2]. Functional disorders and dorsum dissatisfaction are the major causes of revision [3]. They usually occur post-dorsal hump reduction [4]. Thus, they are frequently encountered post-rhinoplasty in the Mediterranean population, where 78% of the patients seek dorsal reduction surgery [5]. Various surgical procedures are discussed in the literature to prevent midvault collapse and to obtain the optimum dorsal aesthetic result [4]. Spreader grafts were first described by Sheen in 1984 to reconstruct the internal nasal valve and prevent postoperative respiratory dysfunction. However, the application of these grafts can be complex, especially in an endonasal approach [6]. Autospreader flaps were initially described in 1950, first utilized in 1990, and constantly modified by multiple surgeons [7]. They were described in closed and open nasal approaches [8]. We present our modified technique of autospreader flaps in endonasal rhinoplasty, and the long-term patient reported a functional outcome. This study was presented at the 67th Congress of the French Society of Plastic, Reconstructive, and Aesthetic Surgery.

## Materials and methods

We conducted a retrospective study for patients operated on by endonasal rhinoplasty with autospreader flaps at the Lebanese University Hospital in Geitaoui between 2010 and 2020. Ethical approval was obtained from the ethical committee of the hospital.

Our study included patients operated on for primary rhinoplasty or septorhinoplasty using the endonasal approach with autospreader flaps. Exclusion criteria were patients with less than one year of follow-up, psychiatric disorders, and a history of nasal bone fractures.

Medical records were carefully examined. Information regarding age, gender, alcohol intake, smoking history, associated psychiatric disorder, revision rhinoplasty, and causes of revision was obtained.

Eligible patients were then reached by phone. Those who approved our request were asked to give oral informed consent and fill out a questionnaire about the NOSE score [9].

Postoperative nasal obstruction was assessed using the NOSE scale. The NOSE scale is composed of five questions concerning the severity of nasal obstruction. Each item is evaluated using a Likert scale from 0 = not a problem to 4 = severe problem, summarized, and then multiplied by 5, for a total final NOSE score range between 0 and 100. Higher NOSE scores reflect a greater severity of self-reported nasal obstruction. 

All participants were informed about the rationale of the study before answering the questionnaire, and they were informed that participation is voluntary and data recorded anonymously will remain confidential.

Statistics

Data analysis was done using IBM Corp. Released 2019. IBM SPSS Statistics for Windows, Version 26.0. Armonk, NY: IBM Corp. Descriptive analysis was conducted to describe our sample and obtain measures of the frequency of categorical variables. As for the continuous variables, the mean and standard deviation were calculated. The NOSE score was compared between the rhinoplasty and septorhinoplasty groups using chi-squared analysis. A p-value of < 0.05 was considered significant. 

Surgical technique

All the surgeries were performed at the same university hospital by two surgeons with at least 10 years of experience in this technique.

All of the procedures were performed using the endonasal approach under general anesthesia. 

Following the marking of the nose, the next step involves injecting a solution of xylocaine adrenaline for infiltration. Subsequently, intercartilagenous and transfixion incisions are performed.

In cases where septoplasty was not conducted, a submucoperichondreal dissection of the caudal septum was carried out using a freer elevator. Cartilage scissors are then used to separate the upper lateral cartilages (ULC) from the septum in the submucoperichondreal plane.

Hump resection is achieved through component reduction of the dorsum. The dorsal septum is gradually reduced, followed by incremental bone reduction, with special attention given to ULC preservation.

**Figure 1:**
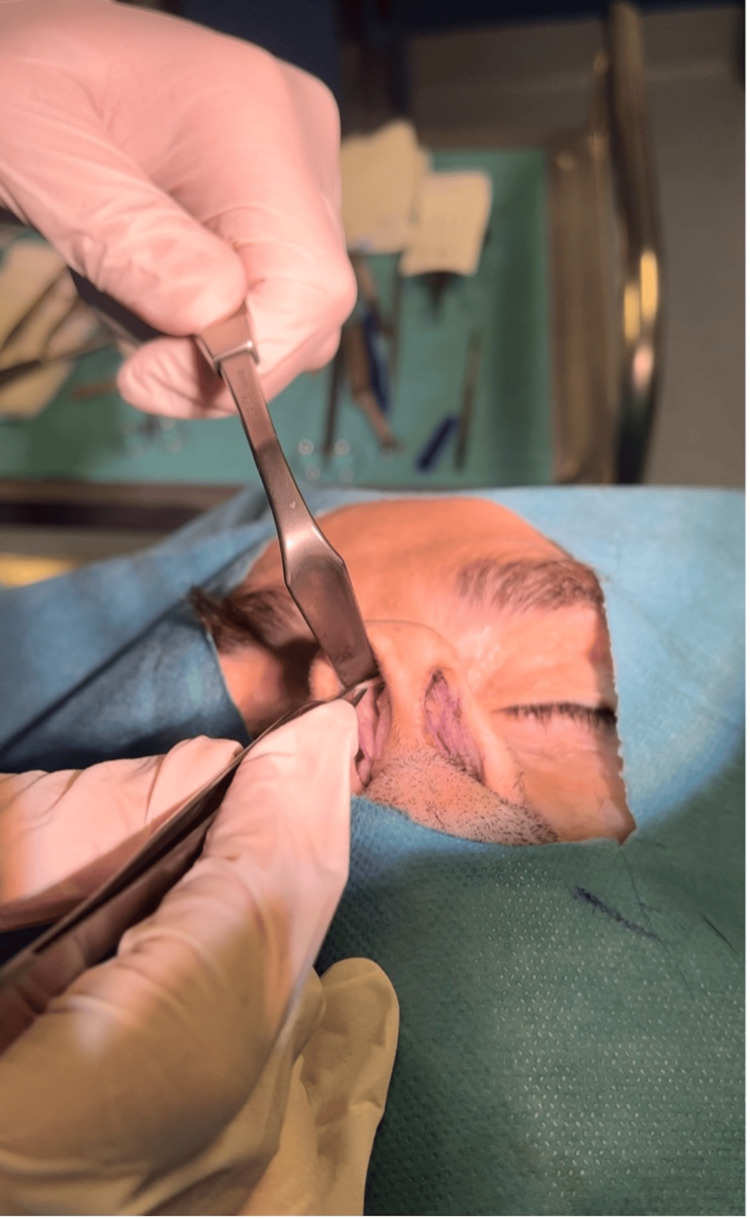
A figure shows the excess ULC after component dorsal reduction. The excess cartilage is incised while keeping the mucoperichondrium intact, creating the spreader flaps

**Figure 2:**
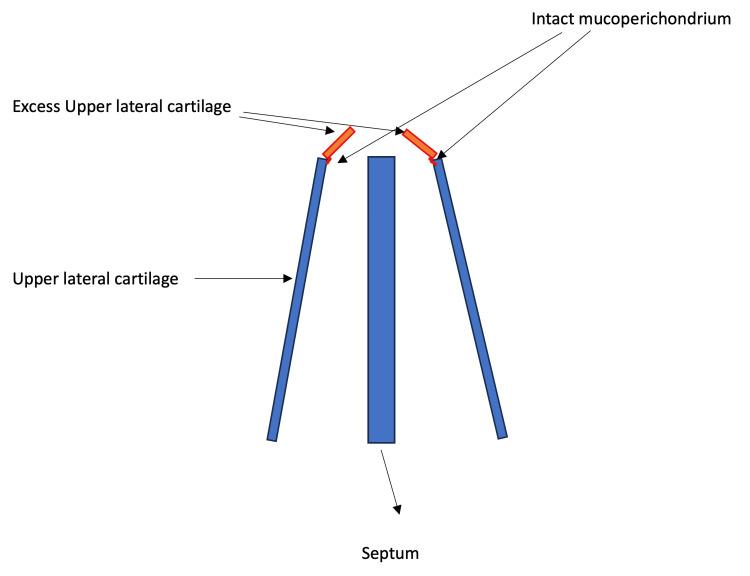
A schematic diagram shows the incision of the excess ULC to create the autospreader flaps while keeping the mucoperichondrium intact. The ULC excess is kept attached to the mucoperichondrium, thus creating the spreader flaps

Autospreader flaps are then created from excess ULC (Figure 1). The surplus ULC is incised while keeping the mucoperichondrium intact (Figure 2).

**Figure 3:**
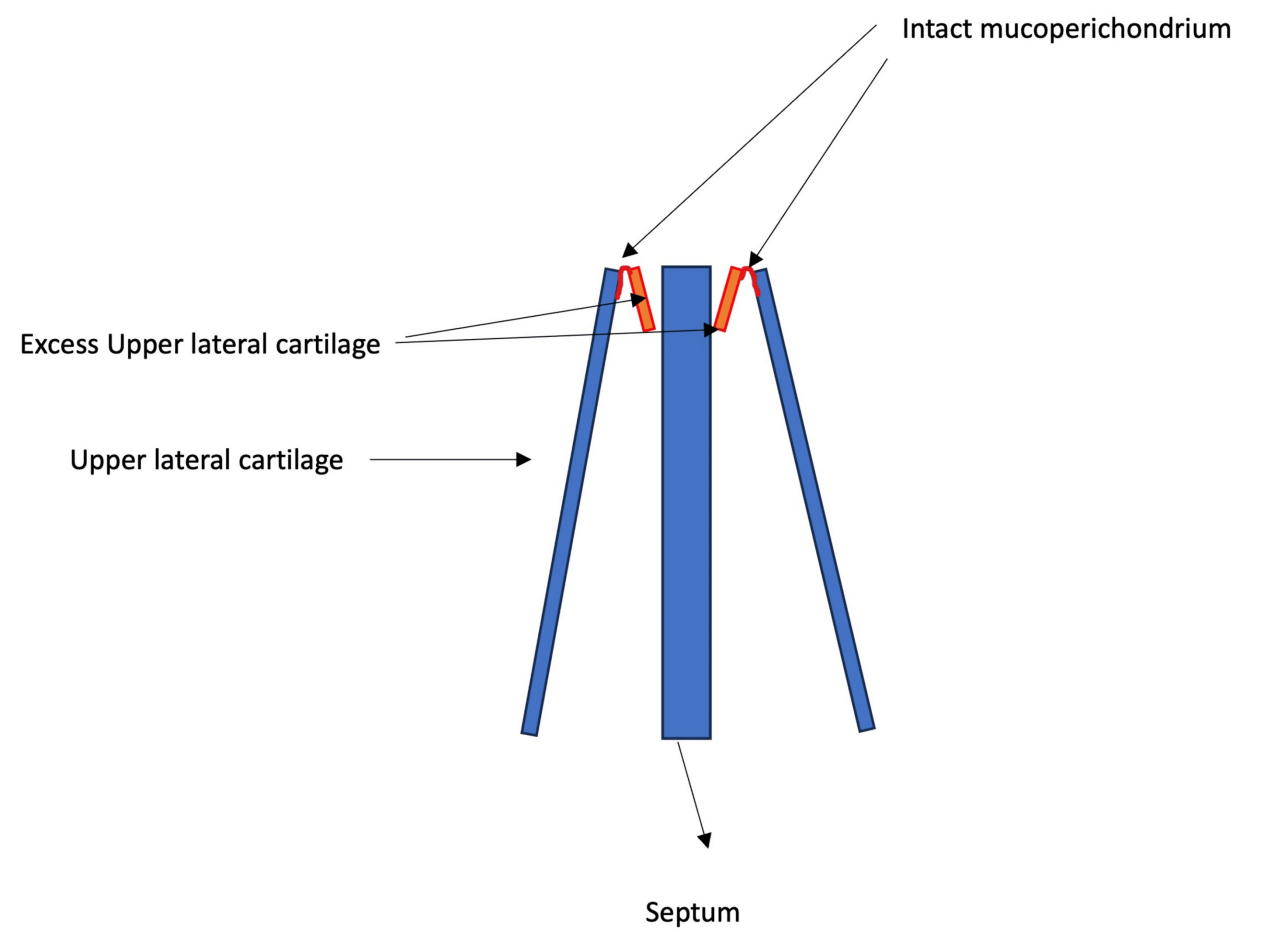
A schematic diagram shows the internal rotation of the spreader flaps. The excess ULC lies between the septum and the ULC to prevent internal nasal valve collapse

**Figure 4:**
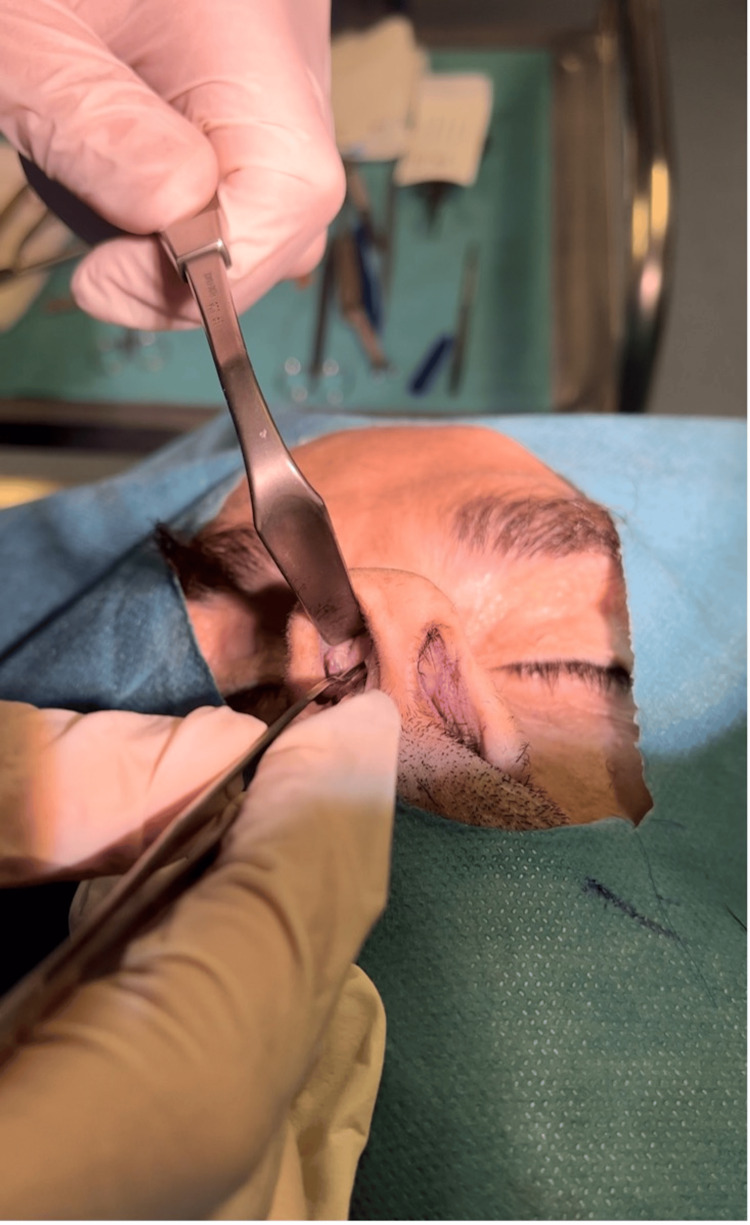
An image shows the autospreader flap lying between the septum and the ULC to reconstruct the middle nasal vault. The mucoperichondrium is kept intact to provide flap stability

Subsequently, this excess is rotated internally to lie between the septum and the ULC (Figures 3, 4).

Cartilage scoring and fixation sutures were intentionally avoided to prevent weakening and fragilization of the ULC.

## Results

**Figure 5:**
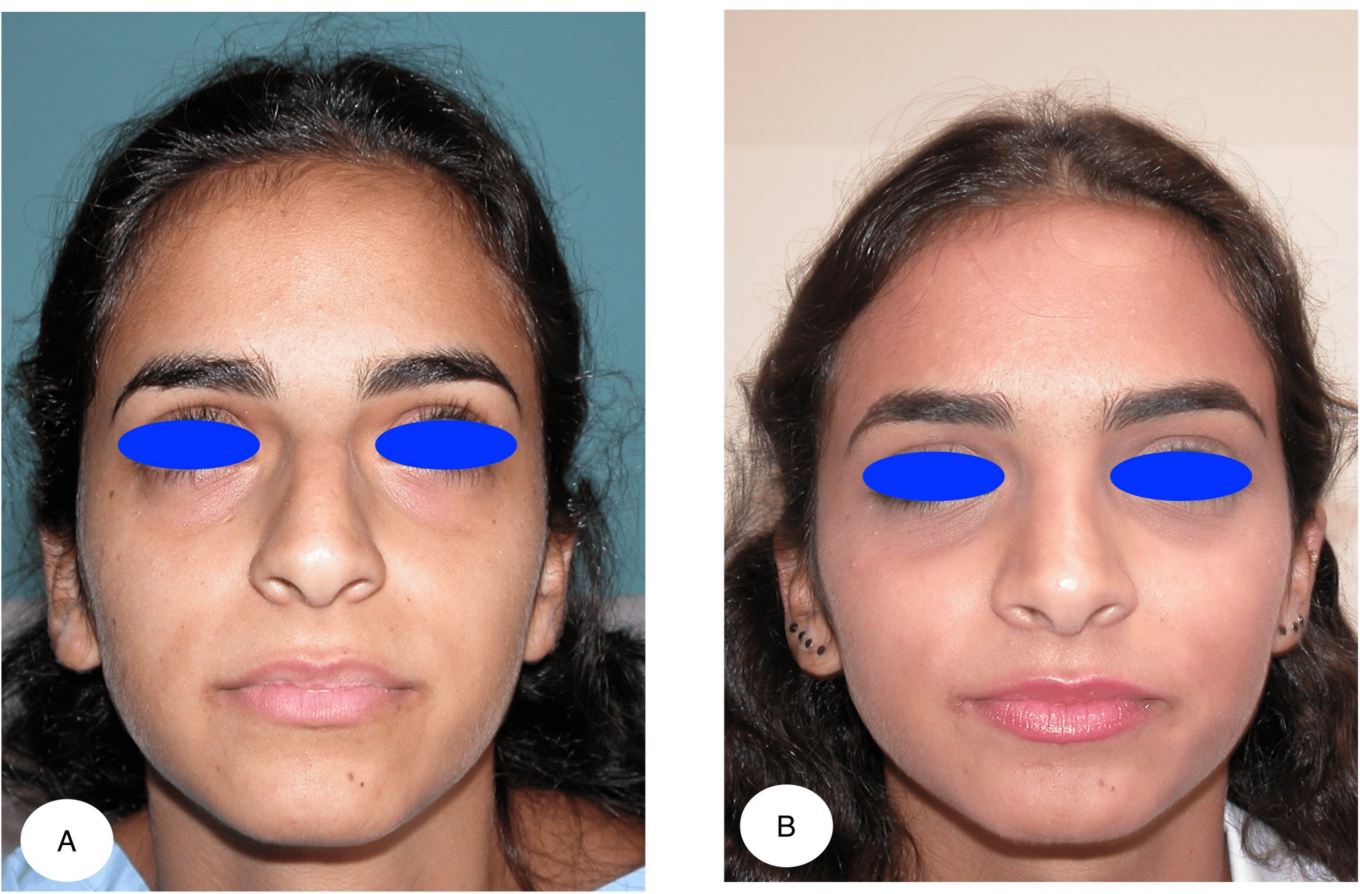
Figure showing the preoperative (A) and postoperative (B) results at one year for a patient operated on using autospreader flaps with an endonasal approach

**Figure 6:**
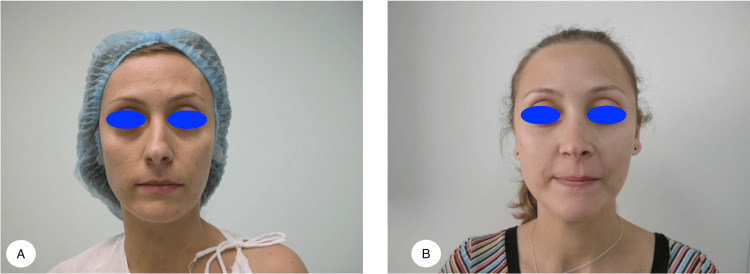
Figure showing the preoperative (A) and postoperative (B) results at two years for a patient operated on using autospreader flaps with an endonasal approach

Our study included 183 patients with a mean age of 26 ± 8.7 years and a mean follow-up of 4.4 years. Twenty-seven of our patients were males, and 156 were females. Satisfactory aesthetic results were obtained (Figures 5, 6).

Only 25 patients required a revision rhinoplasty, with a revision rate of 13.6%. Five patients were reoperated for dorsum dissatisfaction. However, only two patients were reoperated for respiratory difficulties. 

Concerning the NOSE score, it was completed by 87 of the 183 patients, yielding a response rate of 47.5%.

Of the 87 participants, 67 (77%) have had rhinoplasty, and 20 (23%) have had septorhinoplasty. Participant responses to each question on the Nose Scale are presented in Table 1. The mean nose score was 18.1 ± 21.1.

**Table 1:** The NOSE scale checklist of the patients who filled out the postoperative score

Did you experience any nasal blockage or obstruction after the surgery?	53 (60.9%)	12 (13.8%)	13 (14.9%)	7 (8.0%)	2 (2.3%)
Did you experience any trouble breathing through your nose after the surgery?	51 (58.6%)	11 (12.6%)	13 (14.9%)	9 (10.3%)	3 (3.4%)
Did you experience any trouble sleeping after the surgery?	63 (72.4%)	13 (14.9%)	10 (11.5%)	0	1 (1.1%)
Were you unable to get enough air through your nose during exercise or exertion after the surgery?	56 (64.4%)	9 (10.3%)	15 (17.2%)	6 (6.9%)	1 (1.1%)

The results of the Chi 2 test revealed that nasal obstructions were predominantly observed in patients who had rhinoplasty rather than in those who had septorhinoplasty (Table 2). 

**Table 2:** There is a difference in the severity of nasal obstruction between patients operated on for rhinoplasty or septorhinoplasty. The Chi 2 test was used to assess the statistical significance

		Rhinoplasty	Rhinoplasty and septoplasty	p-value
Nasal obstruction	No obstruction	23 (85.2%)	4 (14.8%)	0.351
	Mild obstruction	24 (66.7%)	12 (33.3%)	
	Moderate obstruction	12 (80.0%)	2 (20.0%)	
	Severe obstruction	8 (77.0%)	2 (23.0%)	

However, no significant association between nasal obstruction and the type of surgery was found.

## Discussion

Dorsal hump reduction is commonly sought in rhinoplasty [5]. This reduction can compromise the nasal airways and lead to nasal obstruction [10]. Subjective airway obstruction was reported in 10% of the patients undergoing aesthetic rhinoplasty [11]. Therefore, many techniques were described to reconstruct the middle vault and prevent internal valve collapse. Thus, spreader grafts were first described by Sheen to prevent or treat nasal obstruction post-hump resection. However, ideal positioning and stabilization are complex, resulting sometimes in nasal widening [12]. Their utilization is even more challenging when used in the endonasal approach [13]. Moreover, cartilage harvest is required [6]. Alternatively, spreader flaps can be used to reconstruct the mid-vault. Its usage requires respect for ULC integrity using the component dorsal reduction [14]. This technique is an interesting and reproducible technique in primary rhinoplasty, mainly utilized in the open approach [15,16]. In our practice, we perform the procedure described by Byrd et al.; however, we use the closed rhinoplasty technique, and the ULC is not sutured to the septum [15]. This technique cannot be utilized in all patients. In our experience, we agree with Byrd et al. that its usage is not possible in patients with the associated collapse of bony sidewalls, a deviated dorsal septum, or a deviated nasal tip [15]. We use the autospreader flaps in patients with a high dorsum and an osseocartilaginous hump superior to 2 mm.

Gruber et al. discussed the use of autospreader flaps using the open approach while admitting their difficulty in the endonasal approach. They compared this technique to building a ship in a bottle and suggested omitting the application of a cephalic mattress suture and scoring the flap instead [17]. However, we think that scoring the flaps may weaken the cartilage and may lead to midvault collapse. To add to the effect of narrowing the internal nasal valve by the squeezing of the apical sutures, which may lead to obstructive nasal symptoms [18]. In our practice, turning the ULC excess after the cartilage incision while keeping the mucoperichondrium intact provides midvault stability, enhances the dorsal aesthetic lines, and prevents the widening of the dorsum. This was justified by the fact that only five patients were reoperated for dorsum dissatisfaction. To add to that, only two patients required revision surgery for symptoms of nasal obstruction. Moreover, none of our patients developed an inverted V deformity.

Patient-reported outcome measures have become increasingly essential in modern medical practice to objectively evaluate outcomes [19]. Various postoperative NOSE score values were reported in the literature, with values ranging between 10.9 and 22.1 post-septal deviation surgery [20-23]. A mean NOSE score of 23.1±23.5 was also observed one year post-functional rhinoplasty [24]. In our study, most of our patients did not experience symptoms of nasal obstruction at a mean follow-up of 4.4 years with a mean value of 18.1. Our value falls within the postoperative reported range in the literature. However, our population included patients who underwent aesthetic rhinoplasty and functional rhinoplasty. When analyzing our sample, we found higher NOSE scores in patients operated on for isolated rhinoplasty. However, this difference was not found to be statistically significant. Similarly, Kütük et al. noted no significant difference in NOSE scores at six-month follow-up with respect to the technique used (open or closed), the type of surgery (primary or secondary rhinoplasty), and the indication (functional or cosmetic) [25].

Despite obtaining low revision rates, caution must be exercised when interpreting these results due to the low response rate (47.5%). This rate may introduce response bias, potentially affecting the representativeness and generalizability of the findings. To add to that, the retrospective design of the study with missing data, like the absence of a preoperative NOSE score, may also influence the results. Therefore, while the findings are promising, they should be validated through further studies with higher response rates and prospective designs. 

Thus, in our experience, using autospreader flaps is a simple, reproducible technique with a short learning curve for surgeons specializing in rhinoplasty. It utilizes local tissues to reconstruct the internal nasal valve. It may be easily applied in closed rhinoplasty without suture fixation. The ideal candidates for this technique are patients with straight, non-deviated dorsum and long nasal bones during primary rhinoplasty.

## Conclusions

The autospreader flap is a simple and reliable technique that can be safely utilized in closed rhinoplasty. Encouraging aesthetic and functional results were obtained with low revision rates. Internal valve reconstruction was obtained while preventing dorsal widening. So this technique can be safely applied to well-selected patients, especially in the Mediterranean population.

## Appendices

Nasal obstruction symptom evaluation (NOSE) questionnaire

Please rate the following symptoms over the past 1 month:

1. Nasal congestion or stuffiness

2. Nasal blockage or obstruction

3. Trouble breathing through my nose

4. Trouble sleeping

5. Unable to get enough air through my nose during exercise or exertion

Scoring: 0 = Not a problem 1 = Very mild problem 2 = Moderate problem 3 = Fairly bad problem 4 = Severe problem

Each item score is summed to give a total score, which is then multiplied by 5 to give a score out of 100. Higher scores indicate more severe symptoms.

Concerning figures 2 and 3 no credits/source are available. The two figures were drawn by the authors.
